# Integrated Analysis of ceRNA Regulatory Network Associated With Tumor Stage in Cervical Cancer

**DOI:** 10.3389/fgene.2021.618753

**Published:** 2021-03-23

**Authors:** Xiaojie Ma, Qian Zhang, Jiayu Du, Jie Tang, Bangxian Tan

**Affiliations:** ^1^North Sichuan Medical College, Nanchong, China; ^2^Department of Oncology, Affiliated Hospital of North Sichuan Medical College, Nanchong, China

**Keywords:** cervical cancer, differentially expressed RNAs, biomarkers, competitive endogenous RNA, protein-protein interactions

## Abstract

**Objective:**

To analyze the abnormally expressed genes involved in cervical cancer occurrence and development.

**Materials and Methods:**

Integrated bioinformatics methods were used to analyze differentially expressed (DE) RNAs, including mRNAs, microRNAs (miRNAs), and long non-coding RNAs (lncRNAs), in stage I, II, III, and IV cervical cancer patients from the TCGA database to fully reveal the dynamic changes caused by cervical cancer.

**Results:**

First, DE RNAs in cervical cancer tissues from stage I, II, III, and IV patients and normal cervical tissues were identified and divided into different profiles. Several DE RNA profiles were down-regulated or up-regulated in stage I, III, and IV patients. GO and KEGG analysis of DE mRNA profile 1, 2, 4, 5, 6 and 22 which were significantly down-regulated or up-regulated showed that DE mRNAs are involved in cell division, DNA replication, cell adhesion, the positive and negative regulation of RNA polymerase ll promoter transcription. Besides, DE RNA profiles with significant differences in patient stages were analyzed to perform a competing endogenous RNA (ceRNA) regulatory network of lncRNA, miRNA, and mRNA. The protein-protein interaction (PPI) network of DE mRNAs in the ceRNA regulatory network was also constructed. The network had nine central genes (up-regulated genes: CDKN2A, GSK3B, BIRC5, CYCS, MAD2L1; down-regulated genes: PTEN, FOXO3, CCND2, TGFBR2). Survival analysis found that 5 lncRNAs, 9 mRNAs, and 4 miRNAs can be used as prognostic indicators of cervical cancer. Finally, combined with cluster analysis results, we further screened 2 DE RNAs (AMZ2P1 and HDAC5) using clinical samples, suggesting that AMZ2P1, and HDAC5 may act as diagnostic biomarkers for the development of cervical cancer.

**Conclusion:**

This research provides new effective targets and reliable biological markers for the diagnosis and prognosis of cervical cancer.

## Introduction

Cervical cancer incidence ranks second among female malignant tumors. Globally, there are more than 500,000 new cases and over 260,000 deaths yearly (Stumbar et al., 2019). A large number of studies have shown that high-risk human papillomavirus (HR-HPV) persistent infection is the major cause of cervical cancer (Roden and Stern, 2018). Presently, cervical cancer screening and prevention methods are improving. However, for patients with pathogenic HPV virus, comprehensive treatments results such as traditional surgery, radiotherapy and chemotherapy are unsatisfactory, and patients with advanced-stage have a poor prognosis. Therefore, it is necessary to clarify the molecular mechanism of cervical cancer development and find new key biomarkers for its diagnosis, treatment and prognosis.

Various genes, proteins, and RNA molecules involved in HR-HPV cause the carcinogenesis and progression of cervical cells. These molecules interact with each other to form a complex molecular network, thus boosting cancer progression. Studies have shown that long non-coding RNAs (lncRNAs) can inhibit the down-regulation of microRNAs (miRNAs) on its downstream target genes by combining with miRNAs, thus up-regulating miRNA target genes present. Several miRNAs and lncRNAs interact with each other to form the competitive endogenous RNA (ceRNA) regulatory network in different cancers (Salmena et al., 2011; Tay et al., 2014; Chen et al., 2019). In recent years, it has been revealed that the ceRNA molecular network plays an important regulatory role in promoting the occurrence, development and outcome of cervical cancer (Song et al., 2018; Zhu et al., 2019). There is a ceRNA network of circular RNA in cervical cancer for therapeutic biomarker screening (Gong et al., 2019). lncRNA MIR205HG combined with miRNA 122-5p promote ceRNA regulated cervical cancer cell proliferation and growth (Li et al., 2019).

While many researchers have reported the ceRNA network in cervical cancer (Xia et al., 2018; Du and Chen, 2019; Qin et al., 2019), the molecular mechanism of cervical cancer progression is unknown. Therefore, RNA sequencing data at different cervical cancer stages were downloaded from the cancer genome atlas (TCGA) database to comprehensively understand the ceRNA network during disease progression. In this study, significant differentially expressed (DE) mRNAs, DE lncRNAs and DE miRNAs associated with tumor stages in cervical cancer tissues were screened and identified using bioinformatics methods, which were used for ceRNA regulatory network and PPI network construction. Moreover, survival analysis of DE RNAs in the ceRNA regulatory network was performed for screening candidate miRNAs, mRNAs, and lncRNAs that can affect cervical cancer occurrence and development. Finally, we verified certain analytical results using tissue samples from our own patients.

## Materials and Methods

### Ethics Statement

The 30 paired cervical cancer and adjacent non-tumor tissues were obtained from patients at the Affiliated Hospital of North Sichuan Medical College. All participants signed an informed consent form approved by the ethics committee of the Affiliated Hospital of North Sichuan Medical College prior to inclusion in the study (Ethics number: 2020ER132-1).

### Collection and Screening of RNA-Sequencing Data and Clinical Data From TCGA

The sequencing data of mRNA, miRNA and lncRNA and clinical information for cervical cancer were downloaded from TCGA. TCGA sample information is shown in Supplementary Table 1. The screening criteria for these specimens were: (1) removal of samples that had suffered from other malignant tumors, (2) and those without stage information, and (3) simultaneously organizing the samples using lncRNA, miRNA, and mRNA sequencing data. Finally, 289 cervical cancer samples (158 in stage I, 68 in stage II, 63 in stage III and IV) and 3 normal cervical samples were used for subsequent analysis.

### Acquisition and Cluster Analysis of Differentially Expressed (DE) RNAs

DE mRNAs, DE miRNAs and DE lncRNAs in cervical cancer from tumor-stage I vs. normal, tumor-stage II vs. normal and tumor-stage III and IV vs. normal were screened and analyzed. The mRNAs and lncRNAs with *p* < 0.05 and FDR < 0.05 and FC > 1.5 were considered as DE RNAs, while miRNAs with *p* < 0.05 and FDR < 0.05 and FC > 3 were considered as DE RNAs. The DE RNAs were integrated based on the above DE mRNAs, DE miRNAs, and DE lncRNAs at different stages vs. normal for further analysis. Hierarchical cluster analysis was performed to identify the differential RNA profiles in the normal samples, stage I, II, III, and IV samples. RNA profiles with *p* < 0.05 were considered as DE RNA profiles.

### Gene Ontology (GO) Enrichment and Kyoto Encyclopedia of Genes and Genomes (KEGG) Pathway Analysis of DE mRNA Profiles

The biological function of DE mRNA profiles was systematically and comprehensively analyzed in GO and KEGG database. All functional enrichments and signal pathways associated with DE mRNA profiles were obtained, and the significant functional enrichment and signal pathways were further screened with *p* < 0.05.

### Target DE mRNA and DE lncRNA Prediction

DE mRNAs involved in significant functional enrichment and signal pathways were obtained from GO and KEGG results. miRanda^1^, Targetscan^2^, miRWalk^3^ were used based on the DE miRNAs and the above DE mRNA screening to predict the target DE mRNAs of DE miRNAs. Moreover, miRanda^4^, PITA^5^ were used to predict the target DE lncRNAs of DE miRNAs. Finally, the prediction results of targeted mRNAs and lncRNAs regulated by miRNA were obtained.

### ceRNA Regulatory Network and PPI Network Construction

ceRNA (mRNA-lncRNA-miRNA) regulatory network was constructed using the above-predicted miRNA–mRNA and miRNA–lncRNA interaction data, as follows: (1) the negative correlation pairs of miRNA–mRNA and miRNA-lncRNA were chosen; (2) miRNA–mRNA and miRNA-lncRNA regulatory pairs using Pearson correlation coefficient (PCC) > 0.05 were considered as the candidate regulatory pairs for ceRNA network. In addition, a string database^6^ was used to construct the PPI network of DE mRNAs in ceRNA regulatory network using).

### RNA Extraction and qRT-PCR

We obtained 30 paired cervical cancer and adjacent non-tumor tissues who were diagnosed with cervical cancer based on histopathological evaluation. Total RNA was extracted by using TRIzol reagent (Thermo Fisher Scientific, United States) according to the manufacturer’s protocol. The integrity of RNA was detected by agarose gel electrophoresis. For detection of hsa-miR-142-3p, RNA was reverse-transcribed using miRNA 1st Strand cDNA Synthesis Kit (Vazyme, china), the reverse transcription primer sequence of hsa-miR-142-3p is 5′-GTCGTATCCAGTGCAGGGTCCGAGGTATTCGCACTGGAT ACGACTCCATA-3′, the obtained cDNA was amplified and quantified using miRNA Universal SYBR qPCR Master Mix (Vazyme, china). For lncRNA and mRNA quantifying, cDNA was synthesized using PrimeScriptTM RT Master Mix and detected by SYBR Green qPCR Master Mix kit (Qiagen, Germany) according to the manufacturer’s instructions. β-actin and U6 were used as internal references for quantification of lncRNAs, mRNAs and miRNAs, respectively. All conditions were repeated in triplicate. The 2-ΔΔCt method was used to calculate the relative RNA expression. All primers were purchased from GENEWIZ of China. The primers are below.

AMZ2P1-F: 5′-GCCCAGTTGTGTAGGAGTGA-3′,

AMZ2P1-R: 5′-TTCTGGGGTTGAAGAGGCTG-3′

hsa-miR-142-3p-F: 5′-GCGCGTGTAGTGTTTCCTACTT-3′,

hsa-miR-142-3p-R: 5′-AGTGCAGGGTCCGAGGTATT-3′,

VCL-F: 5′-CTCGTCCGGGTTGGAAAAGAG-3′,

VCL-R: 5′-AGTAAGGGTCTGACTGAAGCAT-3′,

HDAC5-F: 5′-GGTGTGGTCTACGACACGTTC-3′,

HDAC5-R: 5′-GATCCGCTCGCACTTGCTAA-3′.

### Survival Analysis

The uni-factor Cox regression model was used for survival analysis according to survival information and expression data of DE mRNAs, DE lncRNAs, and DE miRNAs in the ceRNA regulatory network. Survival curves of DE mRNAs, DE lncRNAs and DE miRNAs with *p* < 0.05 were considered significant.

## Results

### Identification of DE mRNAs, DE miRNAs and DE lncRNAs Associated With Tumor Stages in Cervical Cancer

**FIGURE 1:**
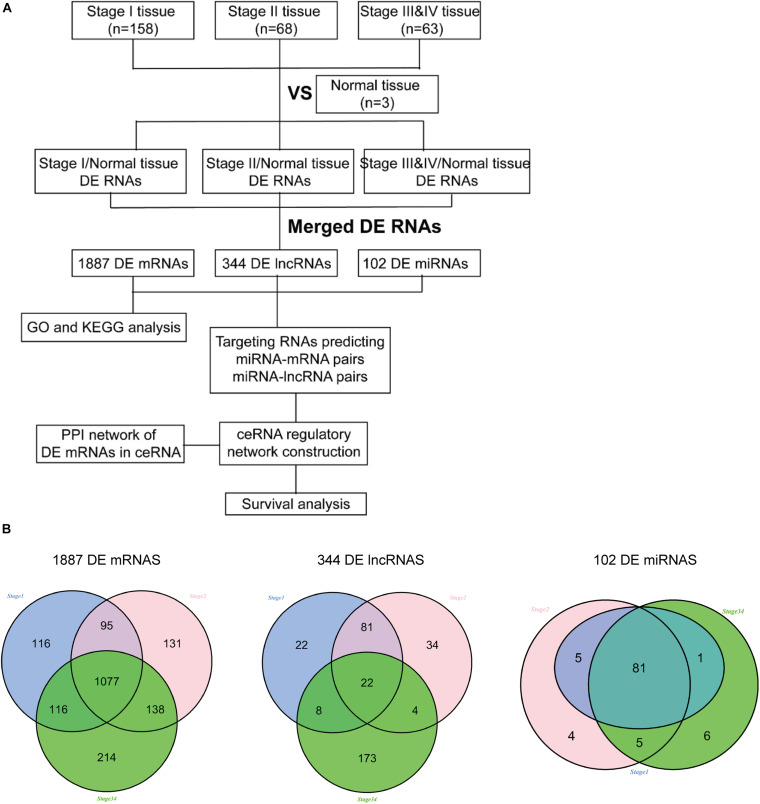
The flow diagram of this study. **(A)** The analysis process of this project; **(B)** Venn diagram of DE mRNAs, DE lncRNAs and DE miRNAs among different stage.

RNA sequencing data from 289 cervical cancer samples (158 in stage I, 68 in stage II, 63 in stage III and IV) and three normal cervical samples were downloaded from TCGA to explore the potential risk of DE RNAs at different tumor stages. Bioinformatics analysis found 1404 DE mRNAs, 133 DE lncRNAs, and 87 DE miRNAs in stage I and normal tissues, 1441 DE mRNAs, 141 DE lncRNAs, and 95 DE miRNAs in stage II and normal tissues; 1545 DE mRNAs, 207 DE lncRNAs, and 93 DE miRNAs in stage III and IV and normal tissues. Finally, these DE RNAs in different stages and normal tissues were merged. A total of 1887 DE mRNAs, 344 DE lncRNAs, and 102 DE miRNAs were obtained for further analysis. The detailed flowchart of this study is shown in Figure 1A. Besides, a Venn diagram was used to show the common and specific DE RNAs at different stages (Figure 1B). The differential and common RNAs information were shown in Supplementary Tables 2–4.

### Cluster Analysis of DE mRNAs, DE lncRNAs and DE miRNAs and Series Test of Cluster

**FIGURE 2:**
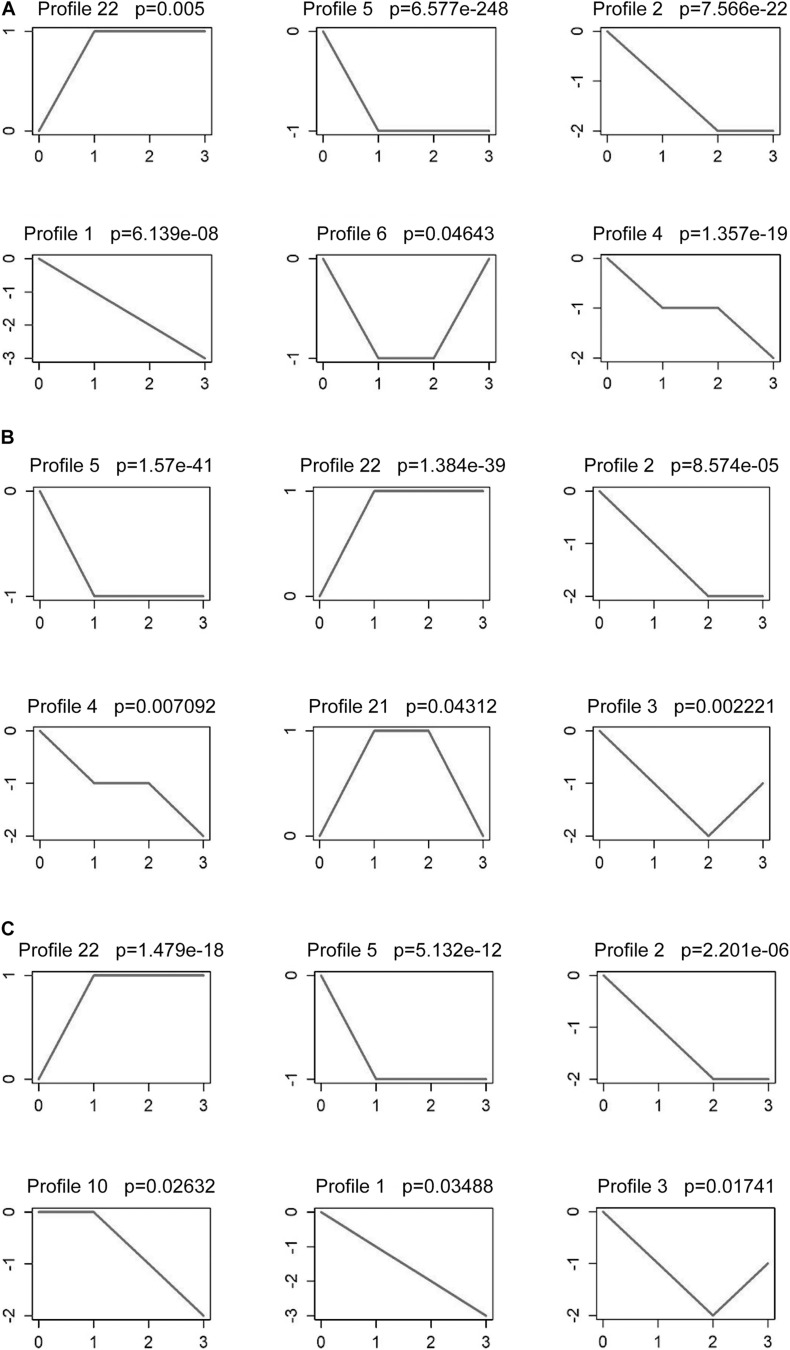
Series test of cluster about DE mRNAs, DE lncRNAs and DE miRNAs. Series test of mRNA profiles **(A)**, lncRNA profiles **(B)** and miRNA profiles **(C)** in stage I, stage II and stage III and IV tissues and normal tissues.

The analysis results showed that the series tests of six mRNA profiles were significant (Figure 2A). mRNA profile 22 showed an upward trend with the cervical cancer development (normal-stage I-stage II-stage III, and IV), and four mRNA profiles (mRNA profile 1, 2, 4 and 5) showed a downward trend. Despite the *p*-value of mRNA profile 6 is less than 0.05, its expression trend was not associated with cancer stages. lncRNA profiles (2, 3, 4, 5, 21, and 22) were significant in tumor development. The analysis results showed that lncRNA profile 22 expression trends increased, while that of lncRNA profiles 2, 4, and 5 decreased as tumor stages increased (Figure 2B). Furthermore, the series tests of miRNA profiles were performed. miRNA profile 22 showed an upward trend and miRNA profiles 1, 2, 5, and 10 showed a downward trend (Figure 2C). Therefore, mRNA profiles (1, 2, 4, 5, and 22), lncRNA profiles (2, 4, 5, and 22) and miRNA profiles (1, 2, 5, 10, and 22) were analyzed to predict tumor progression.

### GO Functional Enrichment Analysis of DE mRNA Profiles

**FIGURE 3:**
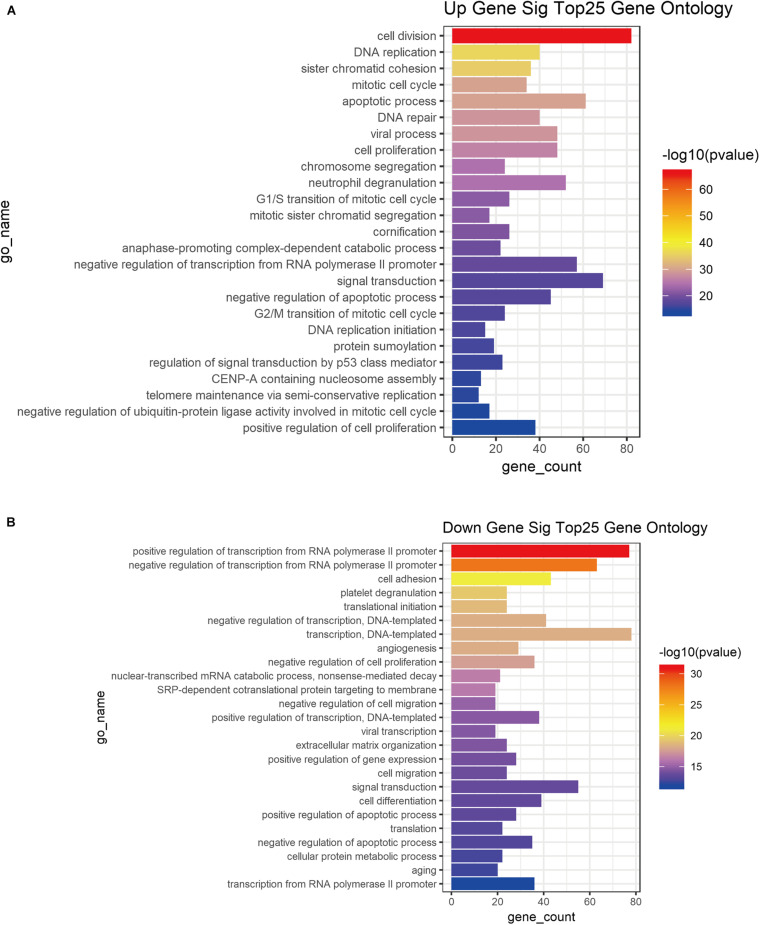
GO functional enrichment of DE mRNAs associated tumor stage. Top25 GO enrichment of up-regulatory **(A)** and down-regulatory **(B)** mRNAs from DE mRNA profiles associated tumor progression.

GO functional enrichment analysis was performed based on the DE mRNA profiles (1, 2, 4, 5, and 22) associated with tumor development to better understand the roles and function of DE RNAs in cervical tumor progression (Figure 3). DE mRNA profiles had multiple functions as the cervical cancer stage progressed. Up-regulated DE mRNA profile 22 was mainly involved in biological processes such as cell division, DNA replication, sister chromatid condensation, mitotic cell cycle, apoptosis, DNA repair (Figure 3A). The down-regulated DE mRNA profiles (1, 2, 4 and 5) were mainly concentrated in biological processes such as positive regulation of RNA polymerase ll promoter transcription, negative regulation of RNA polymerase II promoter transcription, cell adhesion, platelet degranulation, translation initiation, negative transcription regulation (Figure 3B).

### KEGG Pathway Analysis of DE mRNA Profiles

**FIGURE 4:**
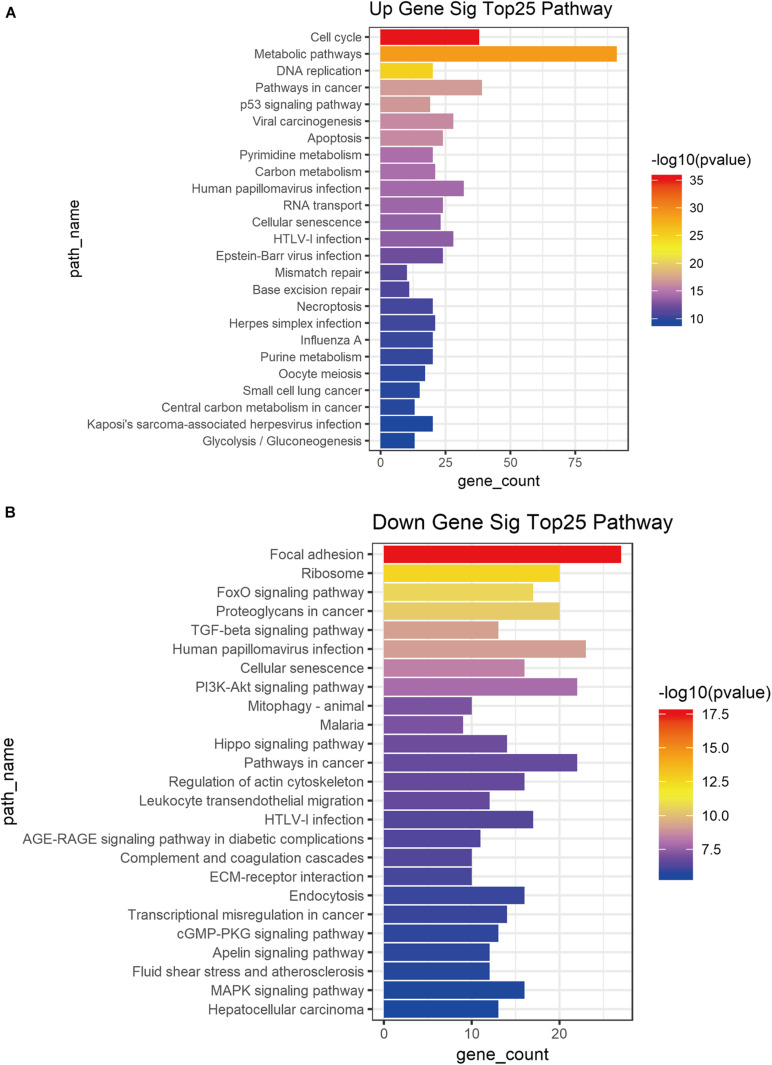
KEGG pathway analysis of DE mRNAs associated tumor stage. Top25 KEGG pathway of up-regulatory **(A)** and down-regulatory **(B)** mRNAs from DE mRNA profiles associated tumor progression.

DE mRNA profiles (1, 2, 4, 5, and 22) were also used for KEGG pathway analysis to demonstrate their function. Cell cycle, metabolic pathway, cancer DNA replication pathway, p53 signal pathway, viral carcinogenesis, apoptosis and other cancer-related pathways were associated with the DE mRNA profile 22 (Figure 4A). The down-regulated DE mRNA profiles (1, 2, 4, and 5) were associated with focal adhesion, ribosome, FoxO signal pathway, proteoglycan in cancer, transforming growth factor-β signal pathway, human baculovirus infection, cell senescence PI3K-Akt signal pathway (Figure 4B).

### ceRNA (mRNA-lncRNA-miRNA) Regulatory Network

**FIGURE 5:**
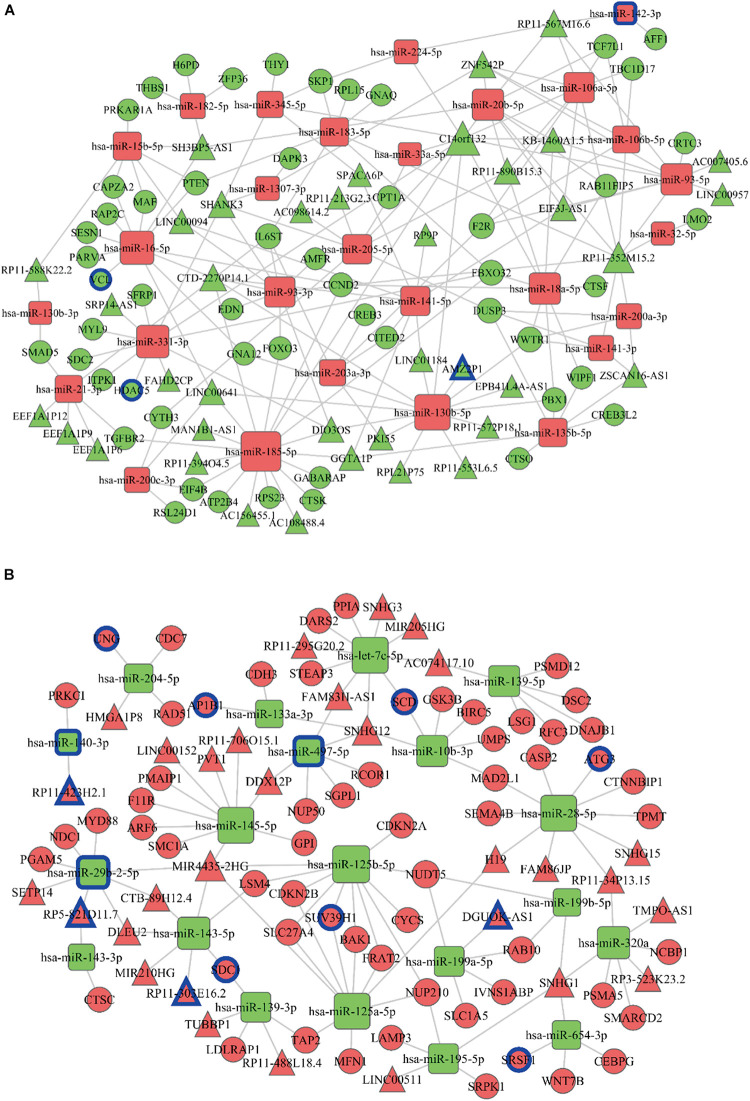
ceRNA regulatory network construction. The ceRNA regulatory network of increased miRNAs **(A)** and decreased miRNAs **(B)** were constructed. The rectangle in the figure represents miRNA, the circle represents mRNA, and the triangle represents lncRNA. Red represents an upward trend with the development of cancer disease; green represents a downward trend with the development of cancer disease; the size of the dot represents the regulatory ability of mRNA. miRNAs, mRNAs and lncRNAs with blue circles are the transcripts predicting overall survival rate.

Combined with cluster series test and DE mRNA profiles involved in top 25 GO functional enrichment and KEGG pathway, 684 DE mRNAs were obtained for ceRNA network construction. First, DE lncRNAs profiles (242 DE lncRNAs) and DE miRNA profiles (94 DE miRNAs) were connected with tumor stages based on 684 DE mRNAs. miRanda^7^, Targetscan^8^, miRWalk^9^ or PITA^10^ were then used to obtain the target DE mRNAs and DE lncRNAs of DE miRNAs to establish ceRNA network. ceRNA network was established using the above results to better comprehend the regulatory molecules of DE mRNAs, including increased miRNAs (Figure 5A) and decreased miRNAs (Figure 5B).

### PPI Regulation Network Establishment

**FIGURE 6:**
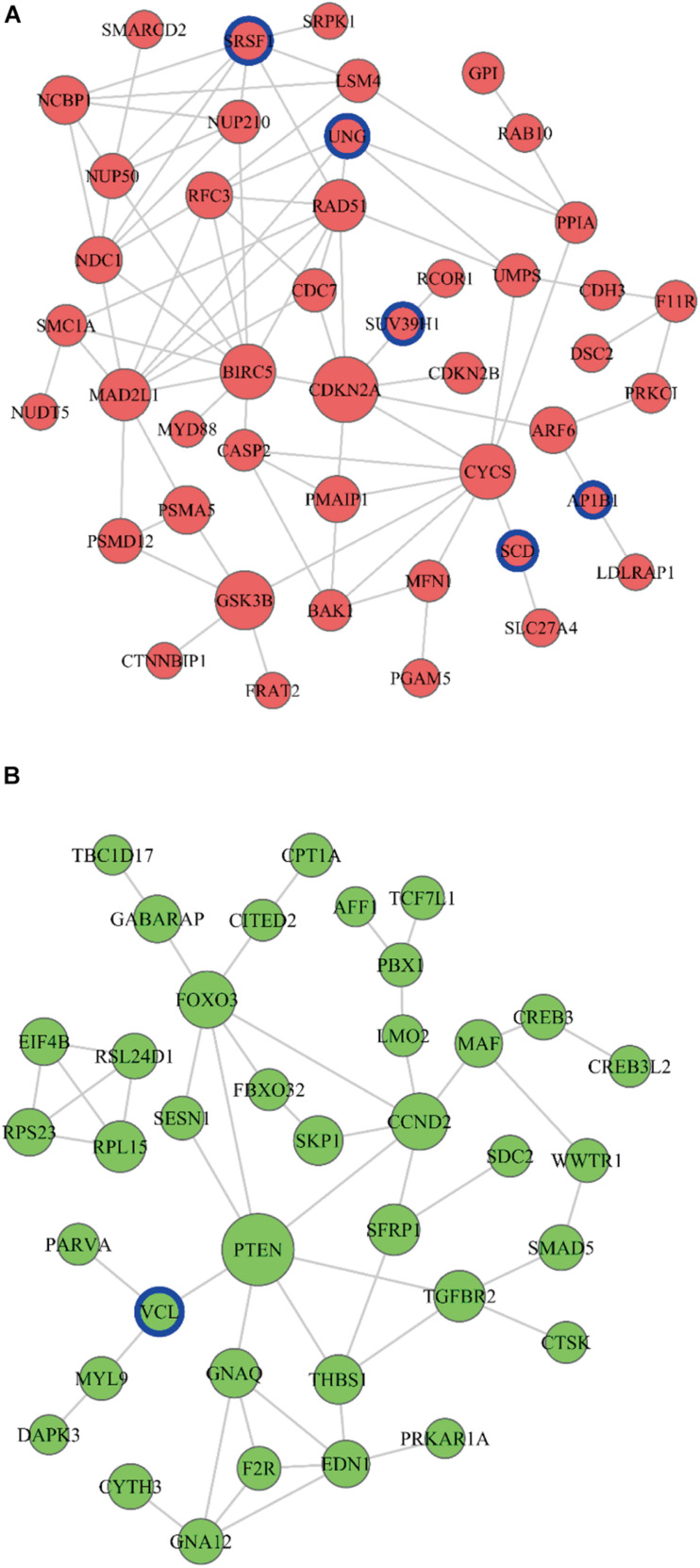
PPI network establishment. The PPI network of increased miRNAs **(A)** and decreased miRNAs **(B)** were established. The circle represents the mRNA, red represents the upward trend with the development of cancer disease, the green represents the downward trend with the development of cancer disease, the size of the dot represents the regulatory ability of mRNA. miRNAs with blue circles are the genes predicting overall survival rate.

A PPI network analysis was performed, and 210 pairs of protein-protein interactions were constructed to further explore the functional implication of DE mRNAs in the ceRNA network. Finally, nine closely related central genes were screened (up-regulated genes CDKN2A, GSK3B, BIRC5, CYCS, MAD2L1; down-regulated genes PTEN, FOXO3, CCND2, TGFBR2) (Figure 6). Moreover, there were six genes (AP1B1, SCD, SRSF1, SUV39H1, UNG and VCL) associated with patient’s survival time in the PPI regulation network (Figure 6).

### Survival Analysis of DE mRNAs, DE lncRNAs, and DE miRNAs in ceRNA Network

**FIGURE 7:**
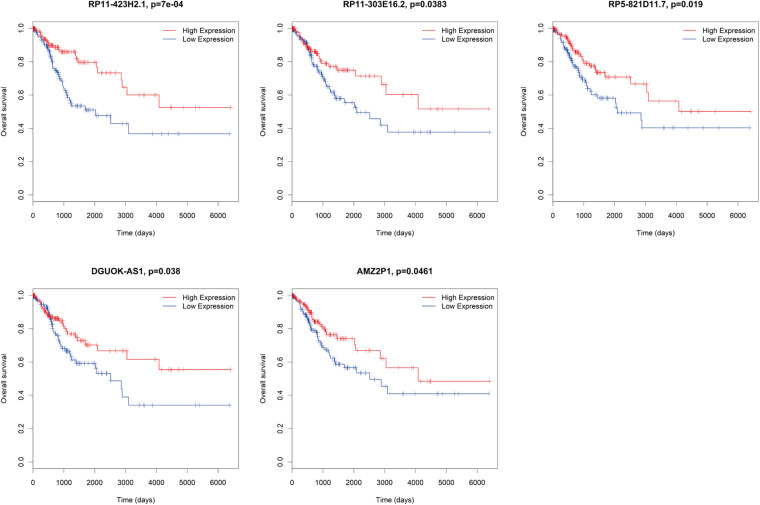
Survival analysis DE lncRNAs in ceRNA network. AMZ2P1, DGUOK-AS1, RP5-821D11.7, RP11-303E16.2, RP11-423H2.1.

**FIGURE 8:**
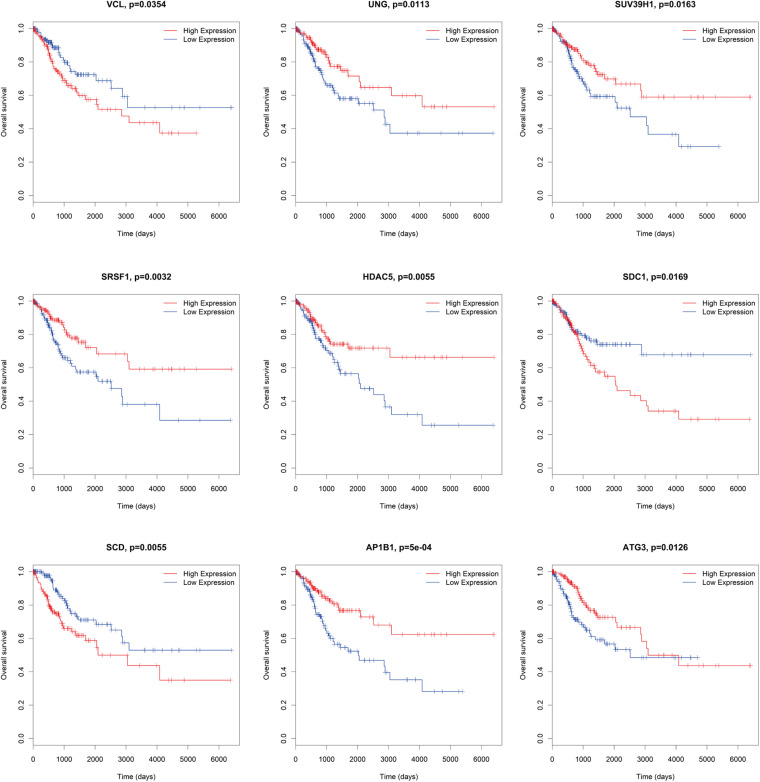
Survival analysis DE mRNAs in ceRNA network. AP1B1, ATG3, HDAC5, SCD, SDC1, SRSF1, SUV39H1, UNG, VCL.

**FIGURE 9:**
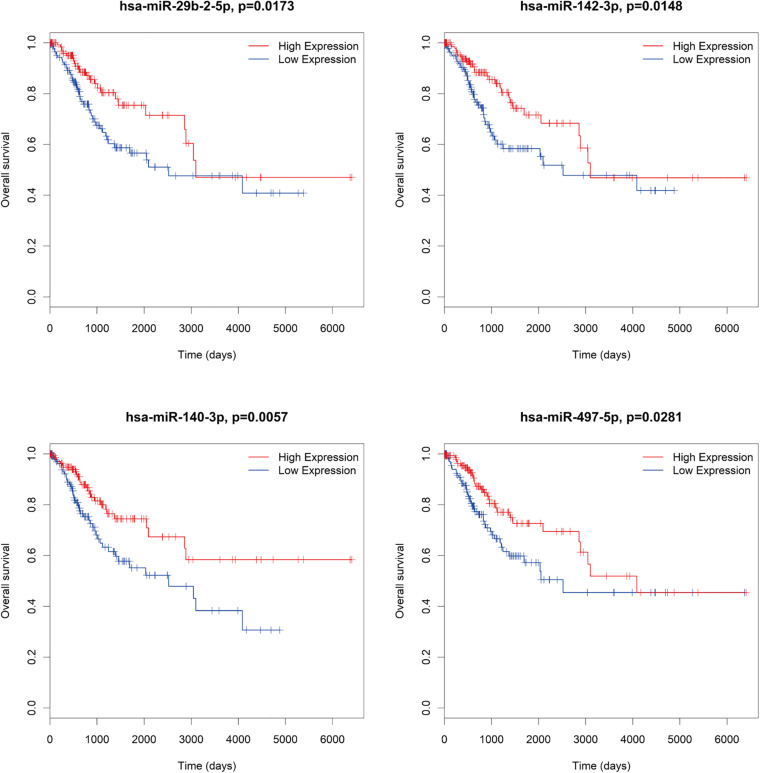
Survival analysis DE miRNAs in ceRNA network. hsa-miR-29b-2-5p, hsa-miR-140-3p, hsa-miR-142-3p, hsa-miR-497-5p.

There were several DE mRNAs, DE lncRNAs, and DE miRNAs involved in cervical cancer progression. Next, survival analysis was performed to evaluate whether these DE RNAs in the ceRNA network predict the prognosis of patients. There were five lncRNAs (AMZ2P1, DGUOK-AS1, RP5-821D11.7, RP11-303E16.2, RP11-423H2.1) (Figure 7), nine mRNAs (AP1B1, ATG3, HDAC5, SCD, SDC1, SRSF1, SUV39H1, UNG, VCL) (Figure 8), and four miRNAs (hsa-miR-29b-2-5p, hsa-miR-140-3p, hsa-miR-142-3p, hsa-miR-497-5p) (Figure 9), which can be used as indexes to predict the prognosis of patients with cervical squamous cell carcinoma. Univariate cox regression analysis of the mRNAs, lncRNAs, and miRNAs are shown in Supplementary Table 5.

### Expression of AMZ2P1, hsa-miR-142-3p, VCL and HDAC5 in 30 Cervical Cancer Patients

**FIGURE 10:**
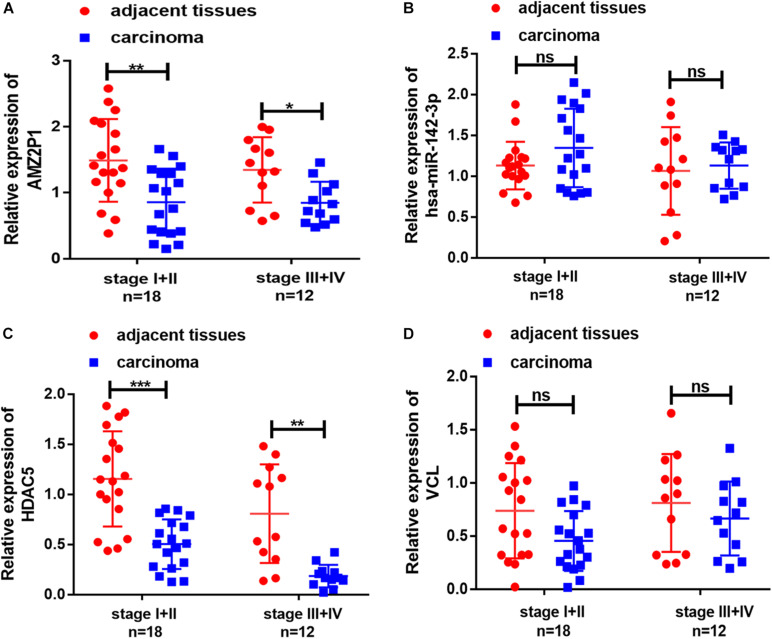
Validation of the expression of representative differentially lncRNA, miRNA and mRNA in cervical cancer tissues and adjacent tissues. The relative expression of AMZ2P1 **(A)**, has-miR-142-3p **(B)**, HDAC5 **(C)**, and VCL **(D)** was detected by qRT-PCR in patient-derived samples with stage I + II (*n* = 18) and stage III + IV (*n* = 12).

Combined with the results in Figure 2, we further screened the 4 DE RNAs (AMZ2P1, hsa-miR-142-3p, VCL, and HDAC5) from 9 DE mRNAs, 5 DE lncRNAs and 4 DE miRNAs closely associated the development of cervical cancer. We investigated the expression of these DE RNAs through qRT-PCR to enhance the reliability of our bioinformatic results. The expression of AMZ2P1 (Figure 10A) and HDAC54 (Figure 10C) was consistent with the results in the ceRNA regulatory network. We also found that there were no difference of hsa-miR-142-3p (Figure 10B) and VCL (Figure 10D) expression between normal and tumor tissues, but the VCL expression in patients with stage III + IV showed an increased trend compared to that in patients with stage I + II, consisted with the survival analysis. Therefore, we were able to verify that AMZ2P1, VCL and HDAC5 exhibited similar expression trends as those predicted using bioinformatic analyses.

## Discussion

Cervical cancer pathogenesis is complex, due to the imbalance of gene expression. In recent years, studies have found that once one or more RNA in the ceRNA regulatory network are abnormally expressed, the homeostasis is destroyed, leading to disease occurrence (Torre et al., 2015). The current study focused on treatment and prediction in early and advanced cancer (Pontes et al., 2020; Shih et al., 2021). Understanding the molecular mechanism is of great significance for better diagnosis and treatment of cervical cancer. 70-gene signature was identified to predict the prognosis in advanced cervical cancer (Nguyen et al., 2020). NF-κB was increased during the progression of cervical cancer (Hua et al., 2019). In our study, we comprehensively investigated the molecular mechanism of the occurrence and development of cervical cancer and found some biomarkers through the study of 289 cases of cervical cancer (158 cases in stage I, 68 cases in stage II, 68 cases in stage III and IV) and 3 cases of normal cervical tissue gene expression profiles, providing new ideas for the diagnosis and treatment of cervical cancer.

The top 25 significant functions and pathways were listed according to the GO functional enrichment and KEGG pathway analysis of DE mRNAs. The results showed that the functions and pathways of DE mRNAs in tumor tissues played an important role in the occurrence and development of cervical cancer. Some studies have found that random errors in DNA replication were the third major cause of cancer, and DNA replication was closely related to the prognosis of cervical cancer. p53 mutation abnormally increases BIRC5 expression levels, while blocking the apoptosis signal pathway, promoting cell proliferation and cell transformation, thus leading to tumor occurrence (Ye et al., 2015; Viloria et al., 2018). p53 expression was decreased in early stage patients with lung cancer (Feldser et al., 2010). The imbalance of CDKN2A, IL1R2, and RFC4 could promote the proliferation of cancer cells, which is closely related to the progress of cervical cancer, and might be a potential diagnostic marker and therapeutic drug targets (Niu et al., 2017). p53/p21/Cdc2-cyclinB1 signal transduction is related to G2/M blockade induced by MTA (Niu et al., 2014). MiRNA 22 inhibited the proliferation and invasion of tumor cells, and promoted apoptosis by down-regulating ACLY (Malvezzi et al., 2018). In the preliminary study of the mechanism of MTA1 promoting the invasion, metastasis and adhesion of cervical cancer cells, it was found that up-regulating the expression of MTA1 in HeLa cells could increase the expression of β-catenin. In turn, it caused the loss of intercellular adhesion and accelerated the invasion, infiltration and metastasis of tumor cells (Restucci et al., 2009). Besides, MMP-9, highly expressed in many tumor cells, is a proteolytic enzyme closely related to tumor metastasis, invasion, and adhesion, and can degrade extracellular matrix, thus promoting cell migration (Tummalapalli et al., 2007; Yamada, 2008). PI3K/Akt signal pathway is an essential signal transduction pathway in cells, playing an important biological role in cell apoptosis, survival, proliferation and cytoskeleton changes. The maladjustment of its regulation was closely related to the occurrence and development of tumor (Chen et al., 2017; Manning and Toker, 2017; Bossler et al., 2019). Researches have demonstrated that cervical cancer development is complex, consistent with this study, which provided theoretical basis for tumor progression.

The interaction between the DE RNAs was analyzed using ceRNA regulatory and PPI networks to better understand the roles of DE lncRNAs, DE miRNAs, and DE mRNAs in cervical cancer development. Nine closely related central DE mRNAs were screened (up-regulated DE mRNAs CDKN2A, GSK3B, BIRC5, CYCS, MAD2L1; down-regulated DE mRNAs PTEN, FOXO3, CCND2, TGFBR2). In addition, we use univariate Cox regression analysis to evaluate the relationship between the expression of DE mRNAs, DE lncRNAS, DE miRNAs in ceRNA regulatory network and patient survival, and found that 5 DE lncRNAs, 9 DE mRNAs, and 4 DE miRNAs could be used as prognostic indicators in patients with cervical squamous cell carcinoma, which were directly or indirectly participated in the development of cervical cancer. Finally, we further screened the 2 DE RNAs (AMZ2P1 and HDAC5) closely associated with the development of cervical cancer by clinical sample. In addition, VCL expression in patients with stage III + IV showed an increased trend compared to that in patients with stage I + II in our study. VCL were also detected in patients with cervical cancer, and VCL expression was associated with TNM stage, this was in line with previous study (Yan et al., 2020).

## Conclusion

In summary, bioinformatic analyses can be used to analyze the core genes and the pathways involved in cervical cancer development. GO and KEGG pathways showed that several DE mRNA profiles participate in the occurrence and development of cervical cancer. Besides, the constructed PPI network showed the interaction between different proteins. DE RNA survival analysis in the ceRNA network verified that these molecules can be used as prognostic indicators. Finally, AMZ2P1 and HDAC5 were identified to be related to prognosis and cervical cancer development using clinical samples.

## Data Availability Statement

The original contributions presented in the study are included in the article/Supplementary Material, further inquiries can be directed to the corresponding author/s.

## Author Contributions

XM and BT designed this study. XM analyzed the data and wrote this manuscript. QZ, JD, and JT downloaded and collected the data. All authors contributed to the article and approved the submitted version.

## Conflict of Interest

The authors declare that the research was conducted in the absence of any commercial or financial relationships that could be construed as a potential conflict of interest.

## Footnotes

1 http://www.microrna.org/

2 http://www.targetscan.org/

3 http://129.206.7.150/

4 http://www.microrna.org/

5 https://genie.weizmann.ac.il/pubs/mir07/mir07_exe.html

6 https://string-db.org/

7 http://www.microrna.org/

8 http://www.targetscan.org/

9 http://129.206.7.150/

10 https://genie.weizmann.ac.il/pubs/mir07/mir07_exe.html

## Supplementary Material

Supplementary Table 1 Clinical information of cervical cancer samples in TCGA.

Supplementary Table 2 Detailed information of mRNAs in Venn diagram.

Supplementary Table 3 Detailed information of lncRNAs in Venn diagram.

Supplementary Table 4 Detailed information of miRNAs in Venn diagram.

Supplementary Table 5 Univariate cox regression analysis of the mRNAs, lncRNAs, and miRNAs.
